# *Campylobacter fetus* meningitis confirmed by a 16S rRNA gene analysis using the MinION nanopore sequencer, South Korea, 2016

**DOI:** 10.1038/emi.2017.81

**Published:** 2017-11-01

**Authors:** Jangsup Moon, Narae Kim, Han Sang Lee, Hye-Rim Shin, Soon-Tae Lee, Keun-Hwa Jung, Kyung-Il Park, Sang Kun Lee, Kon Chu

**Affiliations:** 1 Department of Neurology, Laboratory for Neurotherapeutics, Biomedical Research Institute, Seoul National University Hospital, Seoul 03080, South Korea; 2 Department of Neurology, Seoul National University Healthcare System Gangnam Center, Seoul 06236, South Korea

**Dear Editor,**

*Campylobacter fetus* is a zoonotic pathogen that rarely causes bacterial meningitis in humans.^1^ The majority (>90%) of intestinal campylobacter infections are caused by *Campylobacter jejuni* or *Campylobacter coli*, and only a small portion (~2.4%) are caused by *C. fetus*.^2^ However, *C. fetus* is the most common pathogen that causes *Campylobacter* bacteremia, and the clinical signs of human *C. fetus* infection vary from acute diarrheal illness to systemic illnesses, such as lung abscesses, arthritis and neurological infections.^1^ To date, only ~20 cases of *C. fetus* meningitis have been reported worldwide, mainly in North America and Western Europe.^1, 3^

*Campylobacter fetus* comprises 2 subspecies: *C. fetus* subspecies *fetus* (Cff) and *C. fetus* subspecies *venerealis* (Cfv). Cff has been isolated from the gastrointestinal tracts of sheep and cattle and causes infertility and abortion in these species. By contrast, Cfv is restricted to the genital tracts of cattle, where it causes bovine genital campylobacteriosis.^4^ Most of the *C. fetus* infections in humans are thought to be caused by Cff, but efforts to differentiate the subspecies of *C. fetus* are generally not made. Although Cfv has been isolated from humans in a few cases, its role in human disease is unknown.^2^ A newly proposed subspecies, *C. fetus* subspecies *testudinum,* has been recently isolated from sick humans;^5^ however, its clinical characteristics are largely unknown. Owing to the extremely small number of cases reported as *C. fetus* meningitis, efforts to differentiate the subspecies of *C. fetus* in meningitis patients are lacking. In this study, we demonstrate the first case of *C. fetus* meningitis diagnosed in South Korea, in which Cff was confirmed by 16S rRNA amplicon sequencing on a MinION nanopore sequencer (Oxford Nanopore Technologies, Oxford, UK).

In December 2016, a 64-year-old man with alcoholic liver cirrhosis and poorly controlled diabetes visited Seoul National University Hospital due to fever, nausea and seizure. He had undergone dental implant procedures 2 weeks prior to admission. He reported a 10-day history of upper respiratory infection and decreased oral intake due to gastrointestinal discomfort and recurrent diarrhea. A history of recent travel or animal contact was denied, and his food history was unremarkable.

On day 1, his temperature was 39.2 °C, blood pressure 140/100 mm Hg, heart rate 80 beats/min, and respiratory rate 20 breaths/min. Laboratory tests revealed borderline leukocytosis (10 050 cells/μL) with 79.8% neutrophils, hemoglobin 11.1 g/dL, and a platelet count within the reference range (148 × 10^3^/μL). Oral moxifloxacin was given as an empirical antibiotic; however, on day 3, neck stiffness became pronounced, and a stuporous mental status was noted. Accordingly, a cerebrospinal fluid (CSF) analysis was performed on day 3 and showed pleocytosis (217 leukocytes/mm^3^; 73% neutrophils), a high protein level (188 (reference range 15–45) mg/dL), and a low glucose level (CSF/serum glucose ratio 36.4%). No focal lesions were observed on a brain MRI. Dexamethasone, ceftazidime, vancomycin and ampicillin were administered. *C. fetus* grew in the initial blood cultures but not in the CSF cultures, which were obtained 2 days after empirical antibiotic treatment. The results of other laboratory tests, including viral PCR and serological tests for a wide range of infectious causes of encephalitis, were all negative. Doripenem was administered for 8 days until the patient recovered from the meningitis symptoms without neurologic sequelae. However, the patient was diagnosed with gallbladder cancer during the systemic investigations and was referred to the surgical department for further management. For confirmation of the pathogen, the genomic DNA of the bacteria was extracted from subcultures of a single colony. The pathogen was confirmed as *C. fetus* by the sequence analysis of the full-length 16S rRNA gene using conventional Sanger sequencing.

Additionally, 16S rRNA amplicon sequencing was performed on the MinION nanopore sequencer (Oxford Nanopore Technologies, Oxford, UK). The 16S rRNA genes were PCR amplified from the genomic DNA of the pathogen using the universal bacterial primers 27F 5′-AGA GTT TGA TCM TGG CTC AG-3′ and 1492R 5′-GGT TAC CTT GTT ACG ACT T-3′. Nanopore sequencing libraries were constructed using these amplicons. A total of 43 044 reads were generated during the 51 min sequencing run time. The cloud-based Metrichor 16S-BLAST workflow was applied. Of the 27 173 successfully base-called reads, 26 084 reads were aligned to one of the bacterial 16S rRNA gene sequences. Of the total aligned reads, 26 066 (99.9%) were aligned to the genus *Campylobacter* (Figure 1A). *C. fetus* (23 172 reads, 88.8%) and Cff (15 643 reads, 60.0%) were the top listed species and subspecies, respectively (Figure 1B). The standard methods for the differentiation of *C. fetus* subspecies are tolerance to 1% glycine and H_2_S production,^6^ which were unavailable because the causative strain of this meningitis case had not been stored for further analysis. Instead, the remaining genomic DNA was tested with an additional PCR using previously described primers,^7^ to differentiate the *C. fetus* subspecies. Finally, the pathogen was specified as Cff (Figure 1C).

Only 22 cases of *C. fetus* meningitis have been reported worldwide in adults.^1, 3^ The median age was 48 years (range 23–84 years), and the majority of the patients were men (73%). Sixteen out of the 22 patients were in an immunocompromised state, mostly due to alcoholism (41%) and diabetes (27%). Most of the patients were reported from North America or Western Europe, except for 2 patients reported from Japan.^8, 9^ Although *C. fetus* is a zoonotic pathogen, contact with animals or animal products was identified in <70% of patients.^3^ Food products from cattle and sheep are the most likely routes of transmission in human *C. fetus* infections.^2^ Some studies proposed dental procedures as a possible invasion route for a *C. fetus* infection, and cancer has been suggested as a risk factor for *C. fetus* bacteremia.^10^ The treatment outcome of *C. fetus* meningitis has been favorable, with 17 patients showing full recovery, 3 patients showing a neurologic deficit and 2 deaths.^3^ Several reports have indicated that human *C. fetus* isolates are resistant to ceftriaxone, cefotaxime, penicillin and erythromycin,^3, 11^ while carbapenems are effective.^1, 3^

The MinION is a portable nanopore sequencer that is a third-generation sequencing method. Because it performs low cost, real-time, long-read sequencing, it is increasingly being used for rapid metagenomics analyses of disease/pathogen surveillance.^12, 13^ Real-time surveillance of Ebola was carried out using the MinION.^14^ Recently, remarkable performances of the MinION for rapid bacterial identification by full-length 16S rRNA amplicon sequencing have been reported.^15^ Since deep sequencing of the 16S rRNA gene is possible with MinION, multiple bacterial infections can be detected and their relative abundance can be analyzed by a single sequencing run. However, only a limited number of studies have reported its application in human cases.

This is the first case of *C. fetus* meningitis reported in South Korea, and it is the first to apply MinION-based, full-length 16S rRNA amplicon sequencing in a patient with bacterial meningitis. The patient was in an immunocompromised state with alcoholic liver cirrhosis, uncontrolled diabetes and underlying gallbladder cancer. Although direct animal contact was denied and his food history was unremarkable, he had undergone dental procedures two weeks before admission; this could be the possible invasion route of the pathogen. The patient recovered fairly well after antimicrobial treatment, which included doripenem. The subspecies of *C. fetus* was successfully identified as Cff by 16S rRNA amplicon sequencing using the MinION. We believe this was achievable because of deep sequencing, which enables the identification of small sequence differences in the 16S rRNA gene between two subspecies. Evaluating the efficacy of 16S rRNA amplicon sequencing directly from clinical samples (blood or CSF) is extremely valuable, because it can significantly reduce the turnaround time by omitting the time required for bacterial growth in culture tests. Unfortunately, we could not evaluate this in the current patient because we did not obtain enough specimens before the antimicrobial treatment was initiated.

In conclusion, *C. fetus* should be considered a possible cause of bacterial meningitis, especially in immunocompromised patients with accompanying gastrointestinal symptoms. Nanopore sequencing of the 16S rRNA gene allowed the identification of *C. fetus* at the subspecies level. The capability of differentiating bacterial subspecies makes MinION extremely useful in the epidemiology and surveillance of bacterial infections; however, it should be verified for diverse bacteria obtained from clinical samples in the near future. Nevertheless, the nanopore sequencer would be very useful for pathogen detection in patients with bacterial infections because it enables full-length 16S rRNA amplicon sequencing and the real-time analysis of the reads.

## Figures and Tables

**Figure 1:**
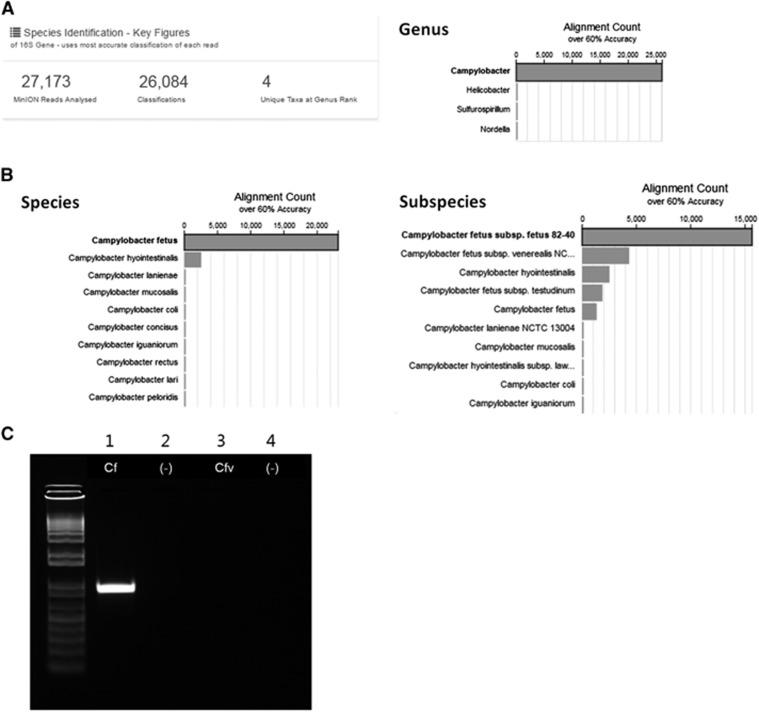
Identification of the pathogen at the species and subspecies level. (**A**) After the MinION 16S rRNA amplicon sequencing, 99.9% of the reads were aligned to the genus *Campylobacter*. (**B**) *C. fetus* (23 172 reads, 88.8%) and *C. fetus* subspecies *fetus* (15 643 reads, 60.0%) were the top listed species and subspecies, respectively. (**C**) Agarose gel electrophoresis of PCR products obtained using primers specific to *C. fetus* (Cf) and *C. fetus* subspecies *venerealis* (Cfv). Lanes 2 and 4 were loaded with a negative control. The band present in lane 1 but absent from lane 3 indicates *C. fetus* subspecies *fetus*.
